# Structural insights into sigma class glutathione transferase from *Taenia solium*: Analysis and functional implications

**DOI:** 10.1371/journal.pntd.0013024

**Published:** 2025-05-30

**Authors:** Ricardo Miranda-Blancas, Ponciano García-Gutiérrez, Cesar Sánchez-Juárez, María C. Cardona-Echavarría, Roberto Flores-López, Rafael A. Zubillaga, Oscar Rodríguez-Lima, Lluvia de C. Sánchez-Pérez, Enrique Rudiño-Piñera, Abraham Landa

**Affiliations:** 1 Departamento de Microbiología y Parasitología, Facultad de Medicina, Universidad Nacional Autónoma de México, Ciudad Universitaria, Ciudad de México, México; 2 Departamento de Química, Universidad Autónoma Metropolitana-Iztapalapa, Ciudad de México, México; 3 Departamento de Medicina Molecular y Bioprocesos, Laboratorio de Bioquímica Estructural, Instituto de Biotecnología, Universidad Nacional Autónoma de México, Cuernavaca, Morelos, México; 4 Posgrado en Ciencias Biológicas, Unidad de Posgrado, Universidad Nacional Autónoma de México, Edificio D, 1° Piso, Circuito de Posgrados, Ciudad Universitaria, Ciudad de México, México; NEHU: North Eastern Hill University, INDIA

## Abstract

Neglected tropical diseases pose a significant threat to global health, especially in low- and middle-income countries where treatment options are inadequate and transmission risk factors persist. One example is neurocysticercosis caused by *Taenia solium.* Sigma class glutathione transferases (Sigma GSTs) are key regulators of Th1 inflammatory responses, making them promising targets for development of therapies and vaccines. This study presents the first report on the crystallographic structures of recombinant 24-kDa sigma class GST from *T. solium* (rTs24GST), which were determined at resolutions of 1.30 and 1.75 Å. The apo-form structures show the typical GST fold with distinct N- and C-terminal domains and highlight regions of notable flexibility near the G-site. Molecular dynamics simulations show that the presence of glutathione stabilizes the enzyme and reduces conformational fluctuations. Comparative analysis with other GSTs revealed conserved flexible regions that correlate with glutathione binding. These structural insights into rTs24GST can be associated with evolutionary adaptations for interacting with diverse substrates and could open new avenues for developing inhibitors and therapeutic strategies against neurocysticercosis.

## Author summary

This study focuses on the structural and functional analysis of a recombinant sigma class 24-kDa GST from *T. solium* (rTs24GST), the worm responsible for human neurocysticercosis, an important neglected tropical disease. Using advanced techniques such as crystallography and molecular dynamics simulations, we determined the high-resolution structure of a rTs24GST, providing new insights into how this enzyme interacts with glutathione, an essential molecule for cellular protection and regulation of immune response. Our findings reveal that the presence of glutathione significantly stabilizes the enzyme, reducing its fluctuations. This suggests that the enzyme’s activity may be influenced by its interaction with glutathione, offering potential avenues for developing new inhibitors or therapeutic strategies targeting neurocysticercosis. Although rTs24GST shows lower activity than other GSTs, its structural flexibility analysis suggests that it may have evolved to interact with a broader range of substrates. This evolutionary adaptation could indicate that this enzyme performs additional functions beyond traditional detoxification processes. These structural insights could prove valuable for the development of new treatments for neurocysticercosis, benefiting affected populations worldwide.

## Introduction

The World Health Organization (WHO) lists 20 neglected diseases that are prevalent in tropical areas that severely affect mainly developing countries [1,2]. Neurocysticercosis is caused by *Taenia solium* and is an example of such diseases present in these countries and developed countries. Currently, there is no commercial human vaccine for *T. solium*, but encouraging progress is being made with a vaccine for pigs [3]. The most effective treatments for cysticercosis are albendazole and praziquantel for humans [4,5] and oxfendazole for pigs, which is effective but associated with secondary adverse effects [6].

Cestodes such as *T. solium* have developed strategies to become established and remain in their hosts for a long time. These mechanisms involve proteins responsible for detoxification, transport, redox homeostasis, and immune response [7–10]. Among the enzymes involved in these processes, cytosolic glutathione transferases (cGSTs) play an important role [11]. The sigma class of cGSTs is also related to these processes, particularly the detoxification of xenobiotics and peroxides due to glutathione-dependent peroxidase activity, as well as immunomodulation of the immune response through the synthesis of prostaglandins [12–14]. In infections by *Schistosoma mansoni*, the sigma class 28-kDa GST (Sm28GST) is crucial in modulating the immune inflammatory response by producing prostaglandin D2 (PGD2), which inhibits the migration of Langerhans cells to the epidermis and leads to the accumulation of dendritic cells in the lymph nodes, which enhances the immune response [15].

Four different classes of cGST have been identified in *T. solium*. A 27.3-kDa GST (Ts27GST) was classified as omega class in the *T. solium* genome project [16]. A 26.5-kDa GST (Ts26GST) is allosteric and the most abundant in *T. solium,* and its structural properties have previously been described for alpha and mu classes [17]. A 25.5-kDa GST (Ts25GST) belonging to the mu class is the least abundant GST in *T. solium* [18]. Ts25GST and Ts26GST are found in parenchyma, protonephridial, and tegumentary cytons; besides immunizing mice with them reduced the cysticerci burden by 90% in a murine cysticercosis model [19,20]. Finally, a 24.3-kDa GST (Ts24GST) of medium abundance, belonging to the sigma class is principally expressed in the scolex and exhibits low activity against 1-chloro-2,4-dinitrobenzene (CDNB) compared to Ts25GST and Ts26GST [21,22].

In the Protein Data Bank (PDB), there are only three crystallographic structures from *T. solium* (PDB IDs 8UJW, 6OOG, and 3MND), and none of them are GSTs. In the presence of praziquantel, significant interactions have been observed near the binding site of glutathione (GSH) in the crystal structure of *Schistosoma japonicum* GST (SjGST, PDB ID 1GTA) [23]. These observations highlight the lack of protein structural information in *T. solium* and underscore the importance of determining new protein structures.

Determining the structures of GSTs from cestodes could uncover critical sites for developing new inhibitors with potential therapeutic use. This paper presents the first report on the crystallographic structure of a recombinant sigma class 24-kDa GST from *T. solium* (rTs24GST) at resolution of 1.30 and 1.75 Å. These structures provide valuable data that could shed light on the dynamic behavior related to the presence or absence of a substrate, which must be considered when developing GST inhibitors.

## Materials and methods

### Protein purification

We followed a previously described method [22]. Briefly, *Escherichia coli* BL21 (DE3) cells (Novagen, Madison, WI, USA) transformed with the pET22b-Ts24GST construct, and were grown in 500 mL of LB medium supplemented with penicillin (100 µg/mL). Protein expression was induced with 0.3 mM isopropyl β-D-1-thiogalactopyranoside (IPTG) when the culture reached an optical density (O.D.) of 0.6. The bacteria were then growth for an additional 4 hours at 37 °C 150 rpm.

The bacterial pellet was harvested by centrifugation at 6,000 *g* for 25 minutes at 4 °C, resuspended, and lysed in 20 mM Tris–HCl buffer pH 7.4 by sonication on ice in the presence of the “cOmplete” protease inhibitor (Sigma-Aldrich, St. Louis, MO, USA). The supernatant obtained after centrifugation was loaded through a column UNOsphere cation exchange resin (Bio-Rad, Hercules, CA, USA). Proteins bound to the column were eluted with a 0-1.0 M NaCl continuous gradient in the same buffer. A second purification step was performed using a Superdex 75 column (GE Healthcare, Chicago, IL, USA) eluted with 20 mM Tris–HCl buffer pH 7.4 and 150 mM NaCl. Fractions of the purification steps were verified by SDS-PAGE.

### X-ray crystallography

rTs24GST was concentrated to 12 mg/mL in 20 mM Tris-HCl at pH 7.4 [22]. Several crystals were obtained using the micro-batch crystallization method in paraffin at 18 °C. Each crystallization test was prepared with 1 µl of rTs24GST and 1 µl of precipitant solution from one of the following crystallization kits: Crystal Screen I, Crystal Screen II, Crystal Screen Cryo, Natrix, Index I, Index II, Quick Screen, pH Screen, PEG/Ion Screen, MembFac, Crystal Screen (Hampton Research, Aliso Viejo, CA, EUA), and Wizard I, II, III, and IV (Rigaku, Charlestown, MA, EUA).

Several rTs24GST crystals were grown in 100 mM sodium citrate solution adjusted to pH 5.8 from the pH Screen kit and chosen for X-ray diffraction analysis. The crystals were flash-cooled in liquid nitrogen in the presence of 40% (v/v) glycerol. X-ray diffraction data for PDB ID 9DDL were collected at 100 K at beamline FMX ID17–2 of the National Synchrotron Light Source II (NSLS2). X-ray diffraction data for PDB ID 9C0A were collected in an X-ray generator at the *Laboratorio Nacional de Estructura de Macromoléculas* (LANEM), UNAM. The diffraction datasets obtained at NSLS2 and LANEM were processed and scaled with XDS [24] and HKL3000 [25], respectively.

Phases were determined using a molecular replacement (MR) implemented in Phaser in the CCP4 suite platform [26]. The template used for the MR was the AlphaFold2 3D model, which was originally generated with ColabFold. The model was improved with a minimization process in Gromacs version 2023 [27] and molecular dynamics (MD) simulations using OPLS force field over a period of 100 ns [28]. Rigid body refinement was performed on the MR coordinates using Refmac 5 into the CCP4 suite [29].

The final refinement process consisted of several cycles until convergence was reached. Alternating manual refinement and building were performed in Coot [30] with an automatic restrained-refinement using Phenix.Refine [31]. The coordinates of the last refinement cycles of both structures were deposited in the Protein Data Bank (RCSB-PDB).

### Molecular dynamics simulations

A 3D structural model of rTs24GST with GSH at the G site was obtained by superimposing its structure with a sigma class GST from *Bombyx mori* complexed with GSH (PDB ID 3VPQ). MD simulations were performed using Gromacs version 2023 [27] with the OPLS-AA force field [28]. A topology ligand file for GSH was generated using LigParGen Tool [32], and the charges were computed with MOE 2014 [33]. First, each rTs24GST (apo) and rTs24GST-GSH (holo) system was analyzed with PropKa [34] to properly protonate amino acidic residues at pH 7.4. The apo and holo systems were solvated with Tip4p-e water and neutralized by adding Na^+^ and Cl^-^ ions to achieve a concentration of 0.15 M.

The steepest descent gradient conjugate algorithm minimized energy for 1000 steps for each system. The solvent and ions were equilibrated at 310 K for 500 ps each in a constant-volume (NVT) ensemble first and then in a constant-pressure (NPT) ensemble. The protein substrate was restrained harmonically using a force constant of 1000 kJ mol^−1^ nm^−1^. Finally, the systems were run in 500 ns MD simulations. The trajectories were recorded for later analysis.

Electrostatic interactions were computed using the particle mesh Ewald (PME) simulation method [35] with a short-range electrostatic cutoff of 1 nm. The short-range cutoff used for Van der Waals interactions during the simulation was also 1 nm. The system was divided into protein and non-protein groups for the temperature coupling using velocity rescaling with a stochastic term. The Parrinello–Rahman method [36] was used for isotropic pressure coupling in the MD simulation with a compressibility of 4.5 × 10^−5^ bar^−1^. Constraints were deployed for all bonds using the LINear Constraint Solver (LINCS) algorithm [37] with a LINCS-order parameter of 4. Three independent replicates were obtained for each system.

### Comparative structural analysis of similar GSTs

We analyzed the mobility of the loops adjacent to the G site in several GSTs crystallized with GSH at that site and its relationship with the affinity towards the substrate. BLAST (https://blast.ncbi.nlm.nih.gov/Blast.cgi?PAGE=Proteins) was used to search for proteins with amino acid sequences similar to Ts24GST, with crystallographic structure deposited in PDB database, and GSH bound to the G site. Only enzymes with reported *K*_*m*_ values for GSH were selected.

The PDB IDs were GSTs from *Fasciola hepatica* (2WB9), *Schistosoma haematobium* (1OE7), *Blattella germanica* (4Q5R), *Drosophila melanogaster* (1M0U), *Nilaparvata lugens* (5H5I), *Bombix mori* (3VPQ), and *Homo sapiens* (6N4E). The B-factor values of the selected PDB IDs were analyzed using UCSF Chimera [38], which were visualized with “*attribute representation”* in a range of 20 (in blue) to 80 Å^2^ (in red). The number and type of interactions between GSH and the G site residues of each GST complex were identified using LigPlot software with a cutoff radius of 3.3 Å [39]. The RMSD calculations were made using UCSF Chimera using Cα for the comparison between all of the structures used.

## Results

**Fig 1:**
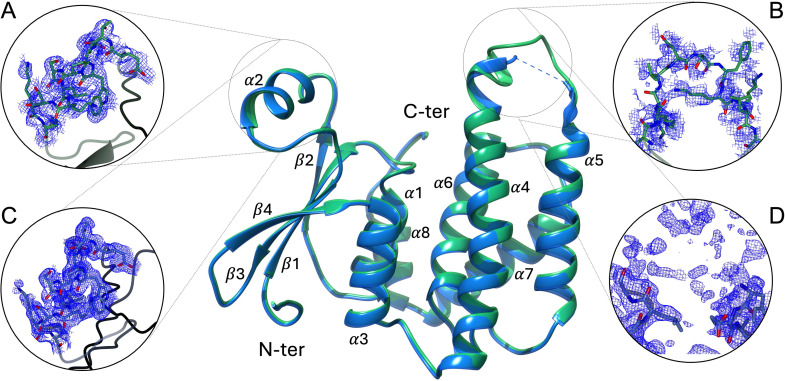
Recombinant Ts24GST crystallographic structures. The central panel shows the superimposed structures of rTs24GST at resolutions of 1.3 Å (blue) and 1.75 Å (green). The electron density maps of the most flexible regions associated with high B-factor values are shown. Panels A and B correspond to the structure at 1.75 Å resolution, while panels C and D correspond to the structure at 1.3 Å resolution. These regions are located in the α2 helix and the loop between the α4 and α5 helices. N-terminal and C-terminal indicate amino and carboxyl terminal domains.

rTs24GST was crystallized under at least 20 different conditions. However, most of the diffracted crystals had low resolution and exhibited multiple lattices or pseudosymmetry problems. The best rTs24GST crystals were obtained in 100 mM sodium citrate at pH 5.8. The phases for the 1.75 Å resolution structure were calculated by the MR method using an AlphaFold model after 100 ns of MD; on this structure, all the aminoacidic residues, including high mobility zones, were constructed using a residual electron density with the aim of using these coordinates for MD calculations in apo and holo forms. The second structure at 1.30 Å resolution was determinated using the rTs24GST 1.75 Å resolution coordinates as the initial model for MR. Moreover, this structure lacks the loop connecting alpha helices 4 and 5 (Fig 1).

The cutoff resolution parameters used were overall values of CC ½ ≥ 0.6, I/σ (I) above 2, and completeness higher than 95%. The crystallographic structure corresponds to the apo form of a sigma class GST. These structures were determined in a C 2 2 2_1_ space group (number 20), and in both cases, the asymmetric unit has a monomer with Wilson B-factor values below 21 Å^2^ (Table 1). The resulting rTs24GST structure has an N-terminalα/β domain (G domain for binding GSH) and an α-helical C-terminal domain (H domain for binding hydrophobic substrates).

**Table 1:** Data collection and refinement statistics.

	Ts24GST 1.3 Å	Ts24GST 1.75 Å
**Wavelength (Å)**	0.97	1.54
**Resolution range**	31.1–1.3 (1.34–1.30)	32.73–1.75 (1.81–1.75)
**Space group**	C 2 2 2(1)	C 2 2 2(1)
**Unit cell (Å)**	57.02 111.3 65.31	56.675 110.113 65.454
**Unit cell (°)**	90 90 90	90 90 90
**V**_**m**_ **(Å**^**3**^**/Da)**	2.14	2.11
**Solvent (%)**	42.45	41.59
**Total reflections**	686969 (65087)	42087 (4102)
**Unique reflections**	50886 (4964)	21048 (2049)
**Multiplicity**	13.5 (13.1)	2.0 (2.0)
**Completeness (%)**	98.97 (97.85)	99.49 (98.51)
**Mean I/sigma (I)**	20.96 (2.64)	33.32 (2.82)
**Wilson B-factor (Å**^**2**^)	15.90	20.69
**R-merge**	0.053 (0.917)	0.030 (0.354)
**R-meas**	0.055 (0.954)	0.043 (0.501)
**R-pim**	0.014 (0.260)	0.030 (0.354)
**CC1/2**	1 (0.88)	1 (0.84)
CC*	1 (0.97)	1 (0.95)
**Reflections used in refinement**	50885 (4964)	20969 (2046)
**Reflections used for R-free**	2588 (241)	1019 (98)
**R-work**	0.18 (0.36)	0.18 (0.30)
**R-free**	0.22 (0.40)	0.22 (0.34)
**CC(work)**	0.96 (0.71)	0.95 (0.72)
**CC(free)**	0.93 (0.77)	0.96 (0.60)
**Number of non-hydrogen atoms**	1979	1969
**Macromolecules**	1784	1797
**Ligands**	7	37
**Solvent**	188	135
**Protein residues**	207	212
**RMS bonds (Å)**	0.006	0.008
**RMS angles (°)**	0.90	1.05
**Ramachandran favored (%)**	96.97	91.90
**Ramachandran allowed (%)**	2.53	5.24
**Ramachandran outliers (%)**	0.51	2.86
**Rotamer outliers (%)**	3.06	2.02
**Clashscore**	6.56	10.63
**Average B-factor (Å**^**2**^)	28.23	36.30
**Macromolecules**	27.59	36.63
**Ligands**	50.23	35.61
**Solvent**	33.48	32.06

Statistics for the highest-resolution shell are shown in parentheses.

Within the asymmetric unit, each rTs24GST monomer consists of 212 residues. The structure of rTs24GST shows a typical GST folding with the thioredoxin domain in the small N-terminal α/β domain (residues 1–90), and the larger C-terminal comprising the α-helix domain (residues 91–212). The N-terminal domain is composed of 3 α-helices (α 1–3) and 4 β-sheets (β 1–4), and the C-terminal domain has 5 α-helices (α 4–8), as indicated in Fig 1.

**Fig 2:**
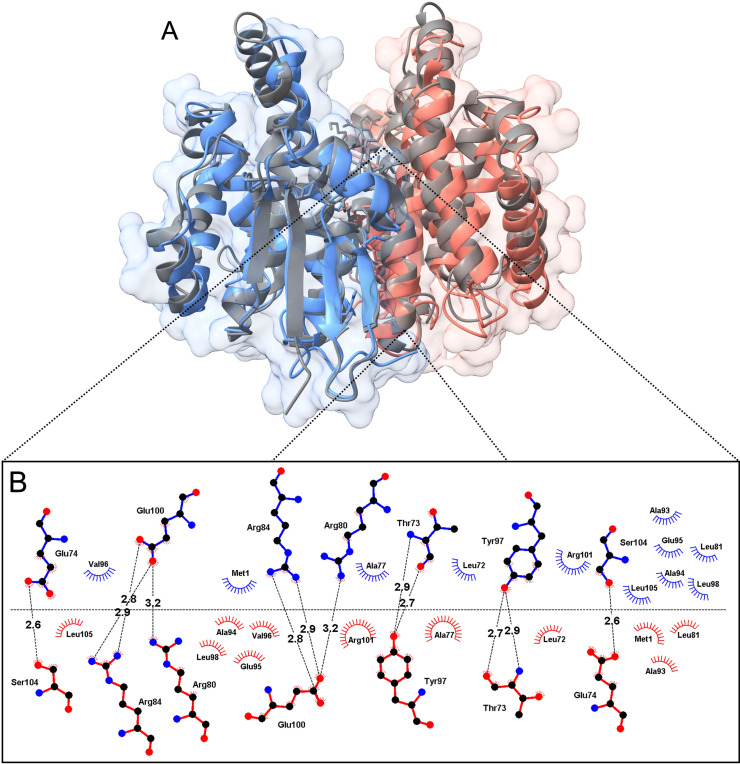
rTs24GST dimer and structural superposition. Dimer structure constructed using symmetry-related operators. Panel A) dimer of rTs24GST (PDB ID 9DDL), each monomer is highlighted in blue and orange colors. In ribbon representation is shown the rTs24GST overlapped with *F. hepatica* crystallographic structure (PDB ID 2WB9 gray color). B) show a zoomed-in view of the dimer interface the amino acid residues and interactions involved in stabilizing the structure.

rTs24GST structure has two regions with high B-factor values, and the first is located between residues Glu42 and Gln56 in the α2 helix. This region shows an electron density high enough for constructing main-chain atoms (Fig 1A and 1C). Moreover, not all side-chains in these residues are associated with a continuous electron density. The second region has the highest B-factor values in the overall structure and is located between residues Leu116 and His132 forming the loop connecting helices α4 and α5. In this region, the electron density is insufficient to build any model (Fig 1B and 1D). The predominant and active form of rTs24GTS is a dimer in solution, confirmed by molecular exclusion chromatography studies [22]. However, in crystals, the biological dimer was constructed using space group symmetry-related operators (https://www.ebi.ac.uk/thornton-srv/databases/pdbsum/). The structural analysis shows 23 residues per monomer involved in the dimeric interface, corresponding to 10.8% of the protein sequence. This interface is stabilized by 10 hydrogen bonds and 10 salt bridges. Structural superposition of rTs24GST (PDB ID 9DDL) coordinates with the crystallographic structure of the GST sigma class from *F. hepatica* (PDB ID 2WB9), considering all C_α_, show an RMSD value of 2.7 Å, which indicates concordance for two orthologous protein structures with only 32.2% sequence identity among them (Fig 2).

The B factor value of GSH contact residues (3.5 Å) in the crystallographic structure of *B. mori* sigma class GST (PDB ID 3VPQ) was compared with those in the structure in the absence of GSH (PDB ID 3VPT). Notably, the mobility of most of these atoms decreases upon binding GSH. For this reason, several attempts were made to determine the crystallographic structure of rTs24GST-GSH complex. However, the diffracted crystals obtained under GSH co-crystallization and soaking conditions did not show the GSH molecule bound to the protein.

To obtain more information in this regard, we performed 500-ns MD simulations on the apo and holo rTs24GST forms. S1A Fig shows the variation of RMSD for both systems, where an increase of up to 0.34 nm can be observed during the first 150 ns in both systems. Subsequently, the values stabilized, and the standard deviation bars indicated fewer fluctuations between replicates in the holo form simulation. Conversely, in the simulation of the apo form, the fluctuations of the RMSD decrease over time.

Both systems’ gyration radius decreased slightly during the first 200 ns of the simulation. The final values of the radius of gyration were marginally smaller for the holo form (S1B Fig). The RMSF analysis of each monomer in the dimer showed lower values for the holo form than the apo form. The highest RMSF values corresponded to residues 110–140 for both apo and holo systems. However, the apo system exhibited larger standard deviations (S2A Fig). Additionally, a difference was observed between the two monomers in the holo form: monomer B had lower RMSF values (S2B Fig); this observation is consistent with the fact that only one monomer is active in the GST dimer.

Principal component analysis (PCA) was performed to identify the main conformations during the trajectories of the dynamics in the apo and holo forms. A critical step and useful feature of PCA is the definition of the number of dimensions to which the dataset can be reduced, which is also known as the essential space dynamics [40]. These appropriate dimensions correspond to the elbow or “weak point” in the graph, from which the eigenvalues decrease constantly. For replicates 1 and 3 of the apo system, this happens in the first 4 components, while for replicate 2, it happens in the first 3.

**Fig 3:**
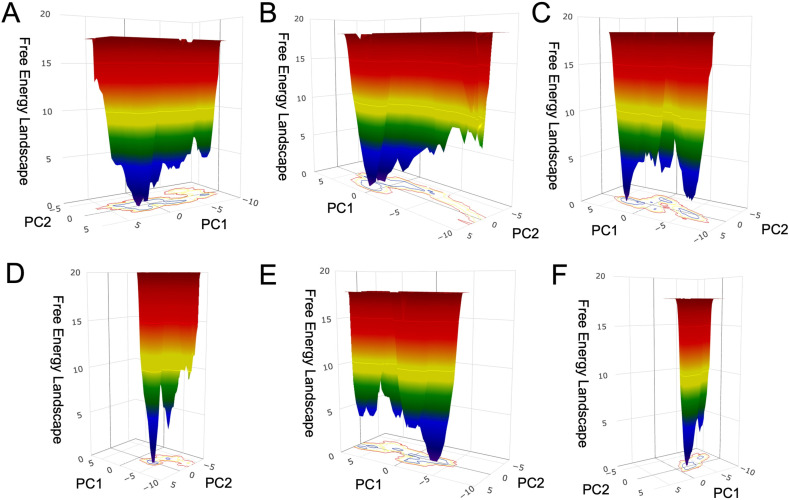
Free-energy landscape of rTs24GST from molecular dynamics simulations with a duration of 500 ns. A triplicate of the MD using an apo state of rTs24GTS is shown above (panels A-C), below are the results with rTs24GTS and GSH as substrate (panels D-F).

However, in the PCA of the MD holo structure, the “weak points” in the graphs appear in the first 3 components for replicates 1 and 2, but they appear in the first 2 components for replicate 3 (S3 Fig). In general, the holo form simulations required fewer components to describe the essential dynamics than the apo form simulations. A 2D projection was performed for the Gibbs free energy landscape on the plane formed by each simulation’s first two essential eigenvectors with the apo and holo structures. The apo system had a more extensive and rugged free energy landscape and number of energy minima (Fig 3A–C) than the holo system (Fig 3D–F).

rTs24GST sequence alignment against the PDB structures revealed 45 PDB entries that belong to 24 different GSTs. Only seven of these proteins had a reported *K*_m_ value (Table 2) [41–47], and only six of those structures had GSH attached to the G site. However, the *F. hepatica* GST had the highest sequence identity regarding rTs24GST, so it was included in the structural analysis despite not having a reported *K*_m_ value.

**Table 2:** Reported values of *K*_m (GSH)_ for rTs24GST and other GSTs.

Protein	Organism	*K*_*m*_ (mM)	Reference
Sigma class GST	*T. solium*	2.05	Sánchez et al., 2024 [13]
Glutathione S-transferase-2	*D. melanogaster*	1.10	Singh et al., 2001 [44]
Glutathione S-transferase	*B. germanica*	0.76	Ma, 2006 [42]
Mu class GST	*S. haematobium*	0.67	Seeletse, 2023 [43]
Hematopoietic prostaglandin D synthase	*H. sapiens*	0.60	Inoue et al., 2003 [41]
Sigma class GST	*B. mori*	0.19	Yamamoto et al., 2013 [47]
Glutathione S-transferase S2	*N. lugens*	0.18	Yamamoto et al., 2015 [46]

**Fig 4:**
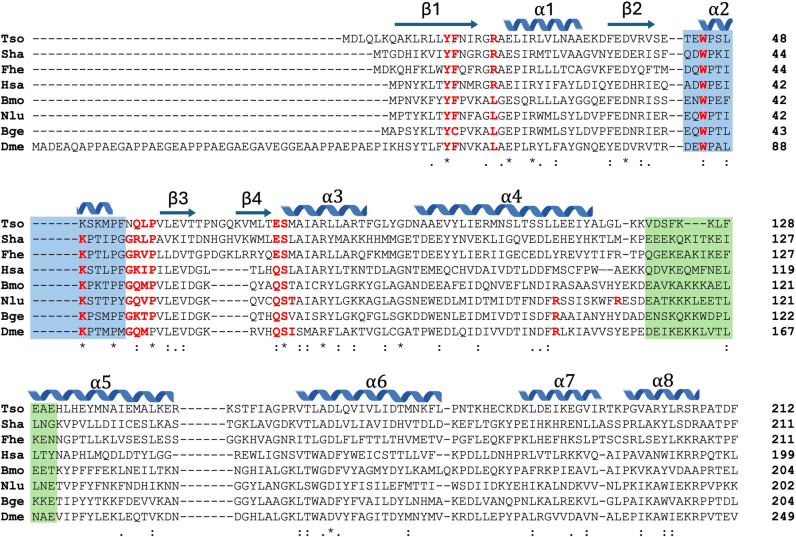
Amino acid sequence alignment of rTs24GST (Tso) with other GSTs whose crystallographic structure is deposited in the Protein Data Bank. Tso, *Taenia solium* ACN88552.1; Sha, *Schistosoma haematobium* P30114; Fhe, *Fasciola hepatica* Q06A71; Hsa, *Homo sapiens* O60760; Bmo, *Bombyx mori* Q5CCJ4; Nlu, *Nilaparvata lugens* J9Q529; Bge, *Blattella germanica* O18598; Dme, *Drosophila melanogas*ter P41043. Residues interacting with GSH are in red, and residues involved in high mobility zones are highlighted in blue and green colors. Symbols indicate fully conserved (*), homolog (:) and weakly similar properties residues (.). Secondary structure is indicated above residues.

Sequence comparison between rTs24GST and the seven previously reported GSTs showed several conserved amino acid residues implicated in GSH interactions in all sequences (Fig 4). Remarkably the *T. solium* complex has the lowest number of contacting residues with GSH. The analysis also revealed low identity in those amino acid sequences corresponding with the zones of high B-factor values (Fig 4, blue and green shading boxes).

**Fig 5:**
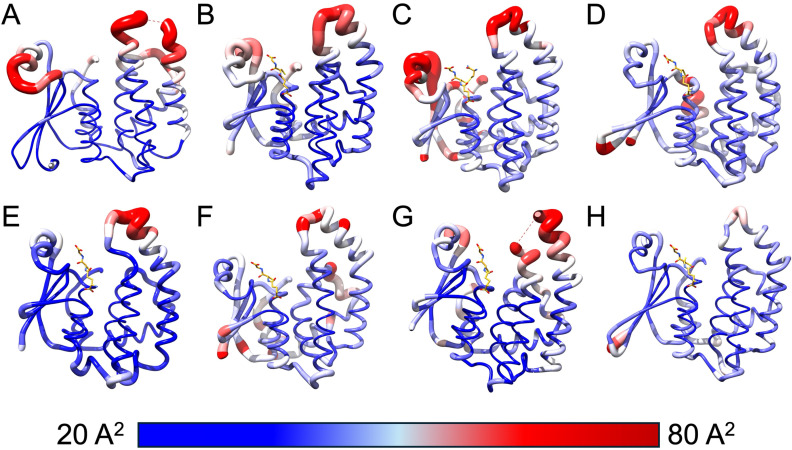
B-factor values representation for GST structures. Comparison of B-factor values between 8 crystallographic structures with most identity to Ts24GST. The representation shows structures from: A) *T. solium*, B) *D. melanogaster*, C) *B. germanica*, D) *S. haematobium*, E) *H. sapiens*, F) *B. mori*, G) *N. lugens*, and H) *F. hepatica.*

The mobile zones identified in the MD analysis of rTs24GST (holo and apo forms) structures correspond to the regions with higher B-factor values in the other seven compared structures (Fig 5A–H). A structural superposition of these structures shows that 103 out of 190 residues analyzed are highly similar (Cα-RMSD = 1.05 Å). The structural similarity is lower when comparing the entire structure (Cα-RMSD = 2.99 Å). The observed differences can be attributed to the loops, which also correspond to the regions with high B-factor values (S4 Fig). Moreover, all the structures revealed that the regions with high mobility consistently overlap, particularly between residues Glu42 and Gln56 near helix α2 and residues Leu116 to His132 between helices α4 and α5 in rTs24GST.

**Fig 6:**
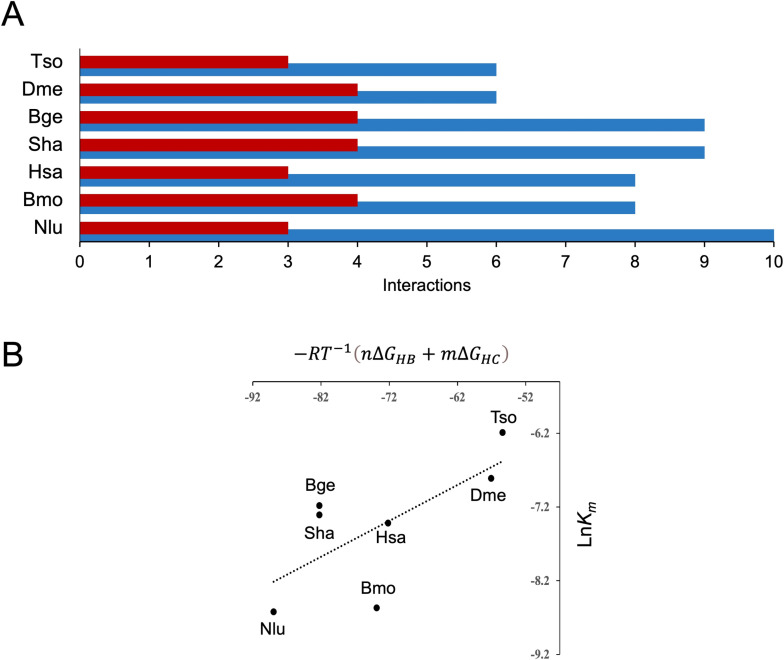
Molecular interactions at the G-site of seven GST-GSH crystallographic complexes and the modeled complex for rTs24GST. A) The number of intermolecular hydrogen bonds and hydrophobic contacts are indicated in blue and red bars, respectively. B) Empirical relationship between the ln *K*_*m*_ value and the mean energetic contribution of hydrogen bonds and hydrophobic contacts between the G-site amino acid residues and the GSH molecule. Tso, *T. solium*; Sha, *S. haematobium*; Hsa, *H. sapiens*; Bmo, *B. mori*; Nlu, *N. lugens*; Bge, *B. germanica*; Dme, *D. melanogaster*.

The rTs24GST-GSH complex was modeled by superimposing the structure of the apo form (PDB ID 9C0A) with the crystallographic complex of the sigma class GST from *F. hepatica* (PDB ID 2WB9). The GST-GSH interactions in the seven PDB entries and the rTs24GST-GSH model were determined by analyzing each complex using LigPlot [39]. The interactions between the modeled rTs24GST-GSH complex and other crystallized GST-GSH complexes were measured to identify the number of interactions within the complex. The analysis shows that rTs24GST has fewer interactions compared to other complexes (Fig 6A), consistent with the lowest number of contacting residues showed in Fig 4.

Assuming that *K*_*m*_ for GSH is equal to *K*_*d*_ (the dissociation constant of the complex GST-GSH) we can write the following equation:


$$\Delta G_{d}=-RTlnK_{m}$$

on the other hand, we express the dissociation free energy as the energy required to break the substrate-enzyme intermolecular interactions. As a first approximation, we consider hydrogen bonds and hydrophobic contacts as the only significant contributors to the binding energy. Assuming average values of 5.0 kcal mol^-1^ and 1.0 kcal mol^-1^ for each hydrogen bond and hydrophobic contact, respectively, we propose the following expression to correlate the number and type of identified interactions with the affinity of the complex.


$$\ln K_{m}=-{RT}^{-1}\left(n\Delta G_{HB}+m\Delta G_{HC}\right)$$

where *n* is the number of intermolecular hydrogen bonds, *m* is the number of hydrophobic contacts, *R* is the gas constant and *T* the absolute temperature. It can be noticed in Fig 6B that the number and type of interactions are correlated with the affinity of the complexes, and that the lowest affinity corresponds to rTs24GST.

## Discussion

rTs24GST is an enzyme with low affinity for GSH, as described previously [21]. This characteristic is evidenced by its inability to bind to a GSH Sepharose-4B matrix [22], unlike other GSTs from *T. solium*, such as Ts25GST and Ts26GST [19]. Nevertheless, this enzyme exhibits low GST activity, similar to Ts25GST but different from Ts26GST and other helminth GSTs [17,18]. To determine which distinctive structural features can explain the relatively low GSH conjugation activity, we analyzed the 3D structure of the GSH-binding site of rTs24GST, and compared it with other GSTs of known structure, and reported *K*_*m*_ values for GSH.

Two crystallographic structures of rTs24GST without GSH (apo form) were determined at resolutions of 1.30 and 1.75 Å and analyzed (Fig 1). However, we could not obtain the crystallographic structure of the rTs24GST-GSH complex (holo form). This may have been due to two different factors. The first relates to the fact that rTs24GST crystallizes with the crystalline organization in different conditions. An analysis of the available space at the G-site in these crystallographic arrangements revealed minimal space for GSH to reach the site due to crystallographic contacts, which reduce or even prevent the success of crystal-soaking experiments with GSH. The second factor is related to the low affinity of rTs24GST for GSH, which hindered the formation of the rTs24GST-GSH complex. These factors suggest that the apo crystalline organization is more stable than the putative GST-GSH complex, and the affinity of rTs24GST for GSH is compromised (at least in the crystallographic conditions tested).

To elucidate the effect of GSH binding to rTs24GST on its structural stability, MD simulations of rTs24GST in the presence and absence of GSH were conducted. The free-energy landscape built with the first two eigenvectors of the PCA analysis showed a less rugged surface and a smaller number of energy minima in the holo-form, in contrast with the behavior of the apo-form (Fig 2). These results suggest that the crystallographic structure of rTs24GST is stabilized in the MD calculations by the presence of GSH at the active site. Similar findings have been reported in previous studies, where several GSTs exhibited more stable states when coupled with GSH [48].

The flexibility and multiple conformational states observed in the MD trajectories in the apo and holo forms of rTs24GST are characteristics observed in other GSTs, and this behavior is not restricted to just one class of GSTs. Additionally, the presence of GSH induces conformational changes at different sites of the enzyme, which in some cases enhance substrate binding at the H-site [49–51]. Similar results have been observed in MD simulation analyses with human alpha-class GST, where the presence of GSH and a substrate at the H-site leads to an enzyme with reduced flexibility and increased stability [52]. Furthermore, mutations at the G-site can enhance not only GSH affinity but also GST activity and stability [53–55].

The comparative analysis highlighted the importance of flexibility and movement in regions associated with GSH binding. Among the seven crystallographic structures analyzed, increased mobility was observed in two specific regions: one in the α2 helix and another between helices α4 and α5. Although these flexibilities are a conserved characteristic, these regions do not have conserved sequences (Fig 3). The primary factor influencing the mobility of these regions appears to be the extent of their interactions with the molecule bound to the G-site. The number and type of interactions between GSH and GSTs, and the reported *K*_m_ values seem to be correlated (Fig 6B). The modeled rTs24GST-GSH complex has six interactions and exhibits the highest *K*_m_ value. In contrast, the number of interactions increases in the other six structures as the *K*_m_ value decreases. The GST with the lowest *K*_m_ showed 10 hydrogen bonds and 3 hydrophobic interactions (Table 2 and Fig 6A). At this point, it is important to note that although there seems to be a trend suggesting a correlation between the number of interactions in the GST-GSH complexes and their affinity (Fig 6B), this is still an approximation that requires further analysis with more accurate computational methods to determine binding free energies.

The results indicate that rTs24GST has unique characteristics that distinguish it from other GSTs. Notably, its low affinity for GSH and the high mobility of regions around the G-site may reflect evolutionary modifications that could enable these enzymes to interact with diverse substrates and even acquire new functions. However, this hypothesis needs to be tested on Ts24GST. Exist some proteins with high sequence similarity to Ts24GST, but their primary biochemical roles are not as GSTs. Rather, they act as prostaglandin synthases whose product (prostaglandin D2) modulates the host’s immune response, or even act as a lens crystallins [44,45,48,56]. Revealing the structure of Ts24GST and its interactions with its substrates could provide insights into its role in cysticercosis and aid in developing possible therapies.

## Conclusion

This is the first report of a GST sigma class crystallographic structure from *Taenia* genus. Crystallographic analysis and MD simulations of rTs24GST, have provided important insights into its structural and functional characteristics. Ts24GST structures in the apo form and rTs24GST-GSH complex (holo) model revealed fewer interacting residues at the G-site that could explain its low affinity for GSH. These findings underscore the possible evolutionary adaptation of Ts24GST to provide diverse functional roles beyond typical GST activity. Furthermore, these findings could contribute in the development of targeted therapeutic strategies against *T. solium*-related parasites.

## Supporting information

S1 Fig Conformation stability in molecular dynamics simulations. rTs24GST simulations in holo (black) and apo (red) form. (A) RMSD values and (B) radius of gyration values of the enzyme.
(PDF)

S2 Fig Root mean square fluctuation (RMSF) of dimeric rTs24GST. The plot shows the chains A) and B) respectively. The color indicates apo in red and holo in black lines.
(PDF)

S3 Fig Eigenvalues for holo and apo systems. PCA results of the top 30 components in Ts24GST molecular dynamics simulations results (A) without GSH and (B) with GSH. The variances (eigenvalues) of each replicate were normalized to facilitate comparison. In the presence and absence of GSH, the linearity of the eigenvalue vs. eigenvector relationship is altered after the first 3 or 4 components, marking the “weak point” in the graphs. This weak point determines the number of essential components to which the data set can be reduced with minimal loss of information. In the six simulations, the first principal component accounted for between 35% and 50% of the total variance, while none of the remaining components contributed more than 15%. The blue, red and black colors correspond to each of the triplicates.
(PDF)

S4 Fig Structural comparison of the rTs24GST with similar GSTs identified in this study. A. Superimposed structures: rTs24GST: green, 1M04 (*D. melanogaster*): fuchsia, 4Q5R (*B. germanica*): purple, 1OE7 (*S. haematobium*): gray, 6N4E (*H. sapiens*): salmon, 3VPQ (*B. mori*): yellow, 5H5L (*N. lugens*): lilac, and 2WB9 (*F. hepatica*): blue. B. RMSD values calculated between rTs24GST and each structure individually, as well as for all structures combined.
(PDF)

S1 Appendix Raw data for the analysis and plot preparations. (XLSX)
