# Supplementary material for: Structural insights into sigma class glutathione transferase from Taenia solium: Analysis and functional implications
Source: PLoS Negl Trop Dis. 2025 May 30;19(5):e0013024. doi: 10.1371/journal.pntd.0013024 (PMC12124585; doi:10.1371/journal.pntd.0013024)
Supplement: S1 Fig — rTs24GST simulations in holo (black) and apo (red) form. (A) RMSD values and (B) radius of gyration values of the enzyme. (PDF) [file pntd.0013024.s001.pdf]

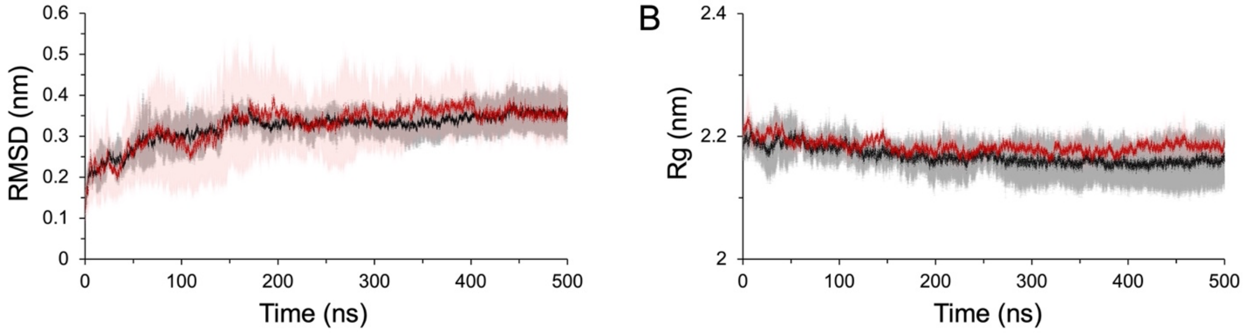

S1 Fig. Conformation stability in molecular dynamics simulations. rTs24GST simulations in holo (black) and apo (red) form. (A) RMSD values and (B) radius of gyration values of the enzyme.
