# Supplementary material for: Structural insights into sigma class glutathione transferase from Taenia solium: Analysis and functional implications
Source: PLoS Negl Trop Dis. 2025 May 30;19(5):e0013024. doi: 10.1371/journal.pntd.0013024 (PMC12124585; doi:10.1371/journal.pntd.0013024)
Supplement: S2 Fig — The plot shows the chains A) and B) respectively. The color indicates apo in red and holo in black lines. (PDF) [file pntd.0013024.s002.pdf]

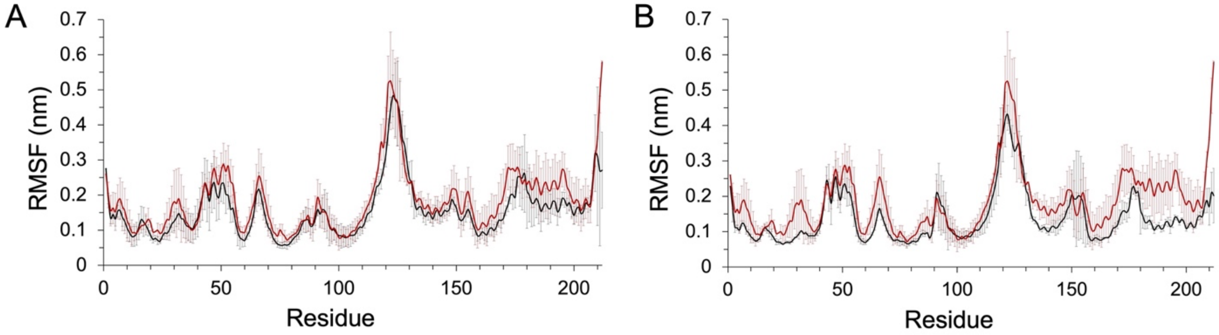

S2 Fig. Root mean square fluctuation (RMSF) of dimeric rTs24GST. The plot shows the chains A) and B) respectively. The color indicates apo in red and holo in black lines.
