# Supplementary material for: Structural insights into sigma class glutathione transferase from Taenia solium: Analysis and functional implications
Source: PLoS Negl Trop Dis. 2025 May 30;19(5):e0013024. doi: 10.1371/journal.pntd.0013024 (PMC12124585; doi:10.1371/journal.pntd.0013024)
Supplement: S3 Fig — PCA results of the top 30 components in Ts24GST molecular dynamics simulations results (A) without GSH and (B) with GSH. The variances (eigenvalues) of each replicate were normalized to facilitate comparison. In the presence and absence of GSH, the linearity of the eigenvalue vs. eigenvector relationship is altered after the first 3 or 4 components, marking the “weak point” in the graphs. This weak point determines the number of essential components to which the data set can be reduced with minimal loss of information. In the six simulations, the first principal component accounted for between 35% and 50% of the total variance, while none of the remaining components contributed more than 15%. The blue, red and black colors correspond to each of the triplicates. (PDF) [file pntd.0013024.s003.pdf]

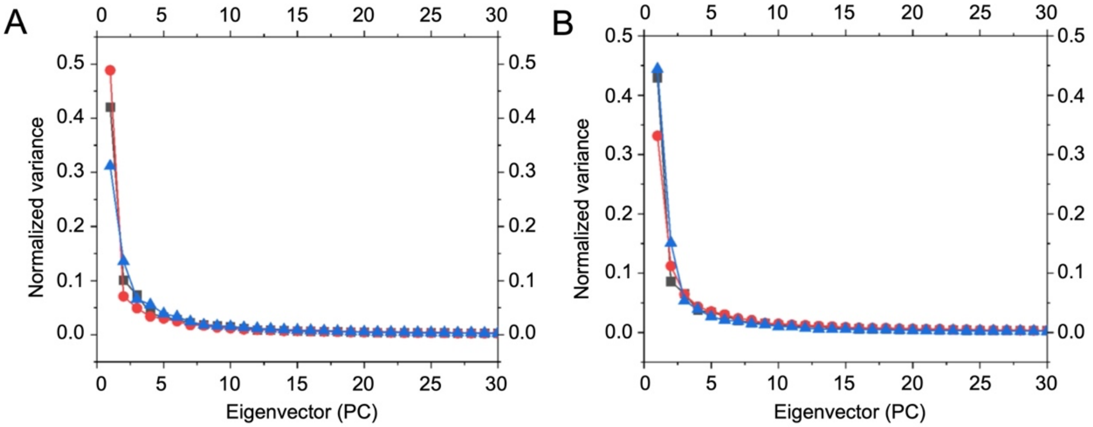

S3 Fig. Eigenvalues for holo and apo systems. PCA results of the top 30 components in rTs24GST molecular dynamics simulations results (A) without GSH and (B) with GSH. The variances (eigenvalues) of each replicate were normalized to facilitate comparison. In the presence and absence of GSH, the linearity of the eigenvalue vs. eigenvector relationship is altered after the first 3 or 4 components, marking the "weak point" in the graphs. This weak point determines the number of essential components to which the data set can be reduced with minimal loss of information. In the six simulations, the first principal component accounted for between 35% and 50% of the total variance, while none of the remaining components contributed more than 15%. The blue, red and black colors correspond to each of the triplicates.
