# Supplementary material for: Structural insights into sigma class glutathione transferase from Taenia solium: Analysis and functional implications
Source: PLoS Negl Trop Dis. 2025 May 30;19(5):e0013024. doi: 10.1371/journal.pntd.0013024 (PMC12124585; doi:10.1371/journal.pntd.0013024)
Supplement: S4 Fig — A. Superimposed structures: rTs24GST: green, 1M04 (D. melanogaster): fuchsia, 4Q5R (B. germanica): purple, 1OE7 (S. haematobium): gray, 6N4E (H. sapiens): salmon, 3VPQ (B. mori): yellow, 5H5L (N. lugens): lilac, and 2WB9 (F. hepatica): blue. B. RMSD values calculated between rTs24GST and each structure individually, as well as for all structures combined. (PDF) [file pntd.0013024.s004.pdf]

A

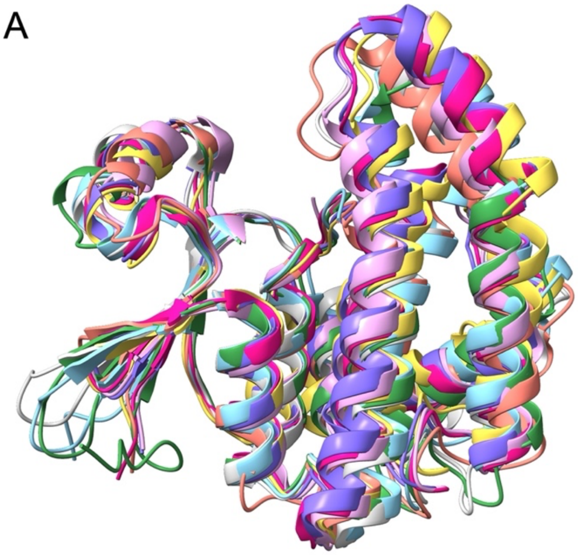

B

PDB ID	RMSD Cα (Å)	Matches	RMSD all Cα (Å)	Matches all
**1M0U**	1.092	123	3.02	191
**4Q5R**	0.938	100	2.90	190
**1OE7**	0.911	139	4.46	189
**6N4E**	1.075	123	3.68	184
**3VPQ**	1.200	109	3.31	192
**5H5L**	1.143	108	3.00	185
**2WB9**	0.953	153	2.46	199
**All structures**	1.053	103	2.988	190

S4 Fig. Structural comparison of the rTs24GST with similar GSTs identified in this study. A. Superimposed structures: rTs24GST: green, 1M04 (*D. melanogaster*): fuchsia, 4Q5R (*B. germanica*): purple, 1OE7 (*S. haematobium*): gray, 6N4E (*H. sapiens*): salmon, 3VPQ (*B. mori*): yellow, 5H5L (*N. lugens*): lilac, and 2WB9 (*F. hepatica*): blue. B. RMSD values calculated between rTs24GST and each structure individually, as well as for all structures combined.
